# CARE Model of Treatment for stuttering: Theory, assumptions, and preliminary findings

**DOI:** 10.3389/fpsyg.2024.1488328

**Published:** 2024-12-10

**Authors:** Courtney T. Byrd, Geoffrey A. Coalson, Edward G. Conture

**Affiliations:** Arthur M. Blank Center for Stuttering Education and Research, The University of Texas at Austin, Speech, Language, and Hearing Sciences, Austin, TX, United States

**Keywords:** stuttering, stammering, theory, treatment, non-ableist, adults, children

## Abstract

The purpose of this article is to present a theory of therapy for stuttering, its related assumptions, and findings from associated empirical studies. Specifically, we propose the Blank Center CARE™ Model of Treatment (CT) for stuttering, which differs from the current, widely employed fluency model of treatment (FT). The CT reflects the authors’ belief in the need to move away from fluency-focused or seemingly ableist treatments (i.e., any approach that attempts to correct, cure, or fix a disabling condition) for stuttering. The authors propose a shift toward a theory of treatment that addresses whole-person wellness and considers the treatment of stuttering from outside the framework of fluency shaping and stuttering modification. In support of such considerations, this article provides preliminary findings from both non-clinical and clinical studies of using the CT for children and adults. Although preliminary, these findings appear to lend empirical support to the authors’ belief that the treatment of stuttering needs to change. In essence, a change in the zeitgeist regarding the treatment of stuttering may contribute to an associated paradigm shift from FT to CT in the management of stuttering in children and adults.

## 1 Fluency treatment

Stuttering represents a multifactorial difference in speech planning and production that typically develops in childhood but continues into adulthood for many individuals. Instances of stuttering are usually typified by repetitions of sounds (e.g., *H-h-he is here*), monosyllabic words (e.g., *He-he-he is here*), and sound prolongations (e.g., *Hhhhe is here*). These disruptions in oral communication are frequently associated with physical tension and effort. Stuttering commonly affects the academic (e.g., Dorsey and Guenther, 2000; Werle and Byrd, 2021), social (e.g., Van Borsel et al., 2011; Zeigler-Hill et al., 2020), and vocational (Gerlach et al., 2018; Klein and Hood, 2004; for workplace discrimination cases, see Andresen v. Fuddruckers, Inc., 2004; Caldera v. Department of Corrections and Rehabilitation, 2020) abilities and activities. The potential emotional and psychological impacts of stuttering due to stigma are often considered a core aspect of the stuttering experience and are included in some contemporary definitions of stuttering (DSM-V, American Psychiatric Association, 2013; Tichenor and Yaruss, 2019). The following will argue, however, that these psychosocial factors need not be considered a defining criterion of stuttering, if the relationship between fluency and communication is proactively prevented from development (i.e., “preventing the iceberg”).

To alleviate stuttering and its related concerns, the treatment with the highest level of evidence—the fluency model of treatment (FT)—targets changing, decreasing, and/or modifying instances of stuttering (see Baxter et al., 2016 and Johnson et al., 2016 for descriptions of treatments). For the purpose of the present manuscript, FT is defined as those treatments that target increasing fluency and/or modifying stuttering. This framework assumes that stuttering compromises an individual’s ability to communicate effectively, putting the onus on the individual to “correct” their “deficiency” (see Milton, 2012 for similar assumptions related to autism). The primary goals of FT appear to be to enhance communication effectiveness by making changes in the frequency, severity, and/or type of stuttering. FT, therefore, assumes that changing stuttering should be associated with changes in communication effectiveness, which should help mitigate internalized stereotyping and increase quality of life (QOL).

Empirical support for this assumption, however, is not robust. Although FT focuses on reducing stuttering, long-term changes are less than apparent based on findings from randomized clinical trials^1^ (RCTs; e.g., Cream et al., 2010; Carey et al., 2010; Carey et al., 2012; Carey et al., 2014; Erickson et al., 2012; Erickson et al., 2016; O'Brian et al., 2003; O'Brian et al., 2008; Menzies et al., 2019a; Menzies et al., 2019b). Specifically, the findings of RCTs indicate that those treated with FT, despite improvements in fluency, find employment of the techniques unnatural-feeling and difficult to maintain (e.g., Arya and Geetha, 2013; Craig and Hancock, 1995; Cream et al., 2003; Irani et al., 2012; National Stuttering Association, 2009; Stewart and Richardson, 2004; Yaruss et al., 2002; see Johnson et al., 2016 for systematic review). Further, such fluent speech is judged by listeners to be no more preferable than moderate-to-high levels of stuttered speech (10–15%; e.g., De Nardo et al., 2023; Manning et al., 1999; Panico and Healey, 2009; Von Tiling, 2011).

Interestingly, FT continues to be a commonly used practice, even though this approach has a longstanding, high relapse rate (60–80% relapse of stuttered speech; Craig and Hancock, 1995), likely due to reported difficulties regarding the naturalness and maintenance of fluency focused techniques (Cream et al., 2003; Yaruss et al., 2002). Empirical findings do not consistently support the notion that FT for adults results in long-term changes in fluency (see Baxter et al., 2016 and Johnson et al., 2016 for systematic reviews). Adults do not necessarily equate the degree to which they can speak fluently with freedom (Venkatagiri, 2009). Likewise, other studies do not support an association between FT and improved communication competence^2^ (speaker-perspective: Constantino et al., 2020; Corcocan and Stewart, 1998; Cream et al., 2003; Plexico et al., 2010; Stewart and Richardson, 2004; listener-perspective: De Nardo et al., 2023; Lee and Manning, 2010; Manning et al., 1999; Von Tiling, 2011) or self-reported QOL (e.g., Boyle, 2015; Byrd, 2021).

Systematic reviews of treatment for stuttering in general indicate that various factors may contribute to a patient’s view of a given treatment’s impact, such as the therapeutic alliance (Johnson et al., 2016). In other words, when the perception of treatment is limited to that of the patient, it is difficult to discern whether the reported impact is specific to the treatment itself or to other unrelated factors. Notably, the present authors have reported (e.g., Byrd et al., 2021; Byrd et al., 2022; Byrd et al., 2024b; Coalson et al., 2024)—based on the perspectives of patients, clinicians, and untrained observers—that changes in fluency are not required for significant changes in the communication effectiveness and/or QOL of children and adults.

### 1.1 Communication as a direct, not indirect, focus of treatment

Among the variety of treatments for stuttering, some focus more on the impact of stuttering than on stuttering itself. These approaches may, therefore, not be exclusively focused on increasing fluency or modifying instances of stuttered speech. For example, Reardon-Reeves and Yaruss (2013) state that the ultimate goal of their therapy for “…children who stutter is to be able to communicate freely…Although improved fluency is a part of this equation, it is not the entire picture” (p. 31). Others report communication benefits when treatment includes stuttering modification or more traditional fluency therapy combined with cognitive behavioral treatment (Blomgren et al., 2005; Kohmäscher et al., 2023; Menzies et al., 2008).

One may argue that all treatments for stuttering target communication, assuming that it will be positively impacted by therapies that focus, at least in part, on increasing fluent speech and/or modifying stuttering. We are not arguing that; instead, we argue for an approach—the Blank Center CARE™ Model of Treatment, or CT—that addresses communication from a different perspective, a pragmatic—rather than fluency—perspective. In particular, the CT directly attempts to strengthen communication competence by improving effective communication (e.g., rate, volume, intonation, gestures, body movement, affect, language use, and organization) across distinct contexts (e.g., speaking to a friend, giving a speech in class, speaking on the phone to set up an appointment), without any direct or indirect attempts to increase fluency. While the FT modifies aspects of communication with the aim of reducing stuttered speech (e.g., changing rate, intonation, and volume) with the expectation of yielding increased fluency and/or modifying individual moments of stuttering, the CT aims to strengthen the communication skills of children and adults such that their overall communication is equally effective as, or more effective than, those who do not stutter, regardless of stuttering frequency. Importantly, the CT strengthens contextual changes in communication skills without discussion, expectation, or requirements for increased fluency or modification to moments of stuttering relative to the usage of these skills. In fact, a core communication skill that is unique to the CT approach is developing and/or strengthening the individual’s open stuttering. As is further outlined in the basic assumptions, the CT does not (in)directly attempt to change stuttering or fluency because the CT model assumes that such attempts compromise the effectiveness of communication and that stuttering openly is fundamental to communicating effectively.

### 1.2 Change in the zeitgeist

Given the above considerations, the authors believe that there is value in rethinking the treatment of stuttering. At present, when the term “strategy” is discussed with respect to stuttering treatment, the prevailing assumption is that it is one that will be used to modify stuttering and/or increase fluency. It is as if the term strategy is exclusively limited to techniques that promote fluency shaping and/or stuttering modification. Thus, any rethinking may constitute something of a shift in the zeitgeist regarding the treatment of stuttering and a shift away from focusing on fluency and ableism,^3^ that is, from attempts to fix the disabling condition, to a focus on whole-person wellness. Of course, such changes in conceptualization remain just that: a change in thinking. For these conceptual changes to meaningfully impact our participants, they need to be instantiated and concretized as something real, specific, and capable of subjecting to scientific scrutiny. To do this, something of a paradigm shift would seem warranted, one that fosters clinical as well as scientific application, experimentation, and study. And, most importantly, such a paradigm shift should involve variables that can be empirically tested, clinically and scientifically, and supported by published empirical findings in peer-reviewed journals.

### 1.3 Need for paradigm shift

The desirability for such a paradigm shift in the treatment of stuttering is reflected by recent changes in field-specific conceptions of stuttering informed by lived experiences (e.g., Byrd, 2021; also see Constantino, 2018; Constantino et al., 2022; Gerlach-Houck and Rodgers, 2022; Watermeyer and Kathard, 2016). These changes parallel the neurodiversity-informed developments in the field of autism research and treatment, wherein clinicians are setting aside neuro-normative expectations in favor of treatment goals that liberate people with autism from the societal pressure to change their natural communication styles for the benefit of the neurotypical listener (see Chapman and Botha, 2023; Elsherif et al., 2022; see also Roberts, 2024). Such a shift is also consistent with the recent focus on ableism (i.e., fixing or curing a disabling condition) in the national healthcare policies of the National Institutes of Health National Advisory Board on Medical Rehabilitation Research [National Institutes of Health-National Advisory Board on Medical Rehabilitation Research (NIH-NABMRR), 2022] and the NIH Advisory Committee Subgroup on Individuals with Disabilities (National Institutes of Health, 2022). This suggested shift is also consistent with the revised policies of the American Psychological Association, which move away from ableist practices during clinical design and practice (American Psychological Association, 2022). These nationwide changes (within the United States) in clinical ideologies provide an opportunity for the field of speech-language pathology to introduce treatment goals beyond those that focus, in part and/or exclusively, on eliminating, reducing, or modifying stuttered speech. Thus, the CT (Byrd, 2023) represents both a theoretical and a therapeutic paradigm shift. This paradigm shift and the CT approach are supported by the authors’ preliminary findings, which will be presented in this article.

### 1.4 Blank Center CARE™ Model of Treatment

As suggested above, we speculate that the lack of consistent empirical support for using FT may be related to one of its basic assumptions. FT suggests that changes in speech fluency are necessary for improved communication and improved QOL. This assumption appears to be challenging to empirically evaluate based on the reported FT methodology. Specifically, it is difficult to assume that fluency is necessary for effective communication by measuring only fluency but not concomitantly determining, measuring, or studying communication itself.

Therefore, it seems that a treatment approach that rests on a different assumption is required. This assumption should also be empirically tested. To meet these requirements, the authors have developed the CT, a model for the treatment of stuttering that has been and continues to be empirically tested, with results disseminated in peer-reviewed journals and discussed below.

### 1.5 Basic differences between FT and the CT

**Figure 1:**
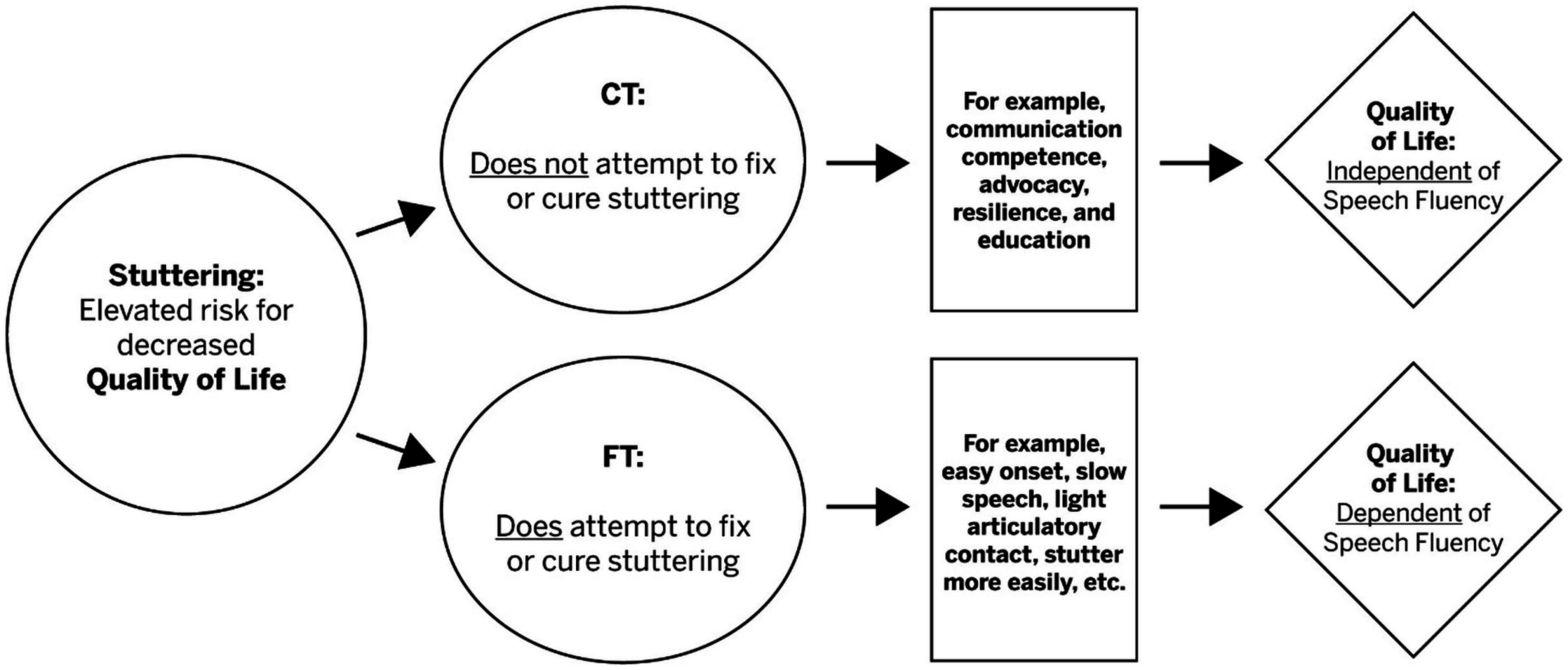
Some essential differences between fluency treatment (FT) and communication treatment (CT) strategies for stuttering treatment.

First, as shown in Figure 1, the CT assumes that the clinical endpoint (far right side of Figure 1) for treating stuttering is a QOL that is *independent* of speech fluency. In contrast, the FT model appears to assume that the clinical endpoint of treating stuttering is a QOL that is *dependent* on fluency, changed, decreased, or modified stuttering.

Second, related to the above assumption, the CT posits that increased fluency and QOL are not synonymous. In contrast, the FT appears to posit that increased fluency (and/or stuttering more easily) and enhanced QOL are synonymous. Indeed, it is an underlying tenet of the CT model that these two clinical goals—any form of targeting decreased stuttering and enhanced QOL—are conceptually incompatible.

**Figure 2:**
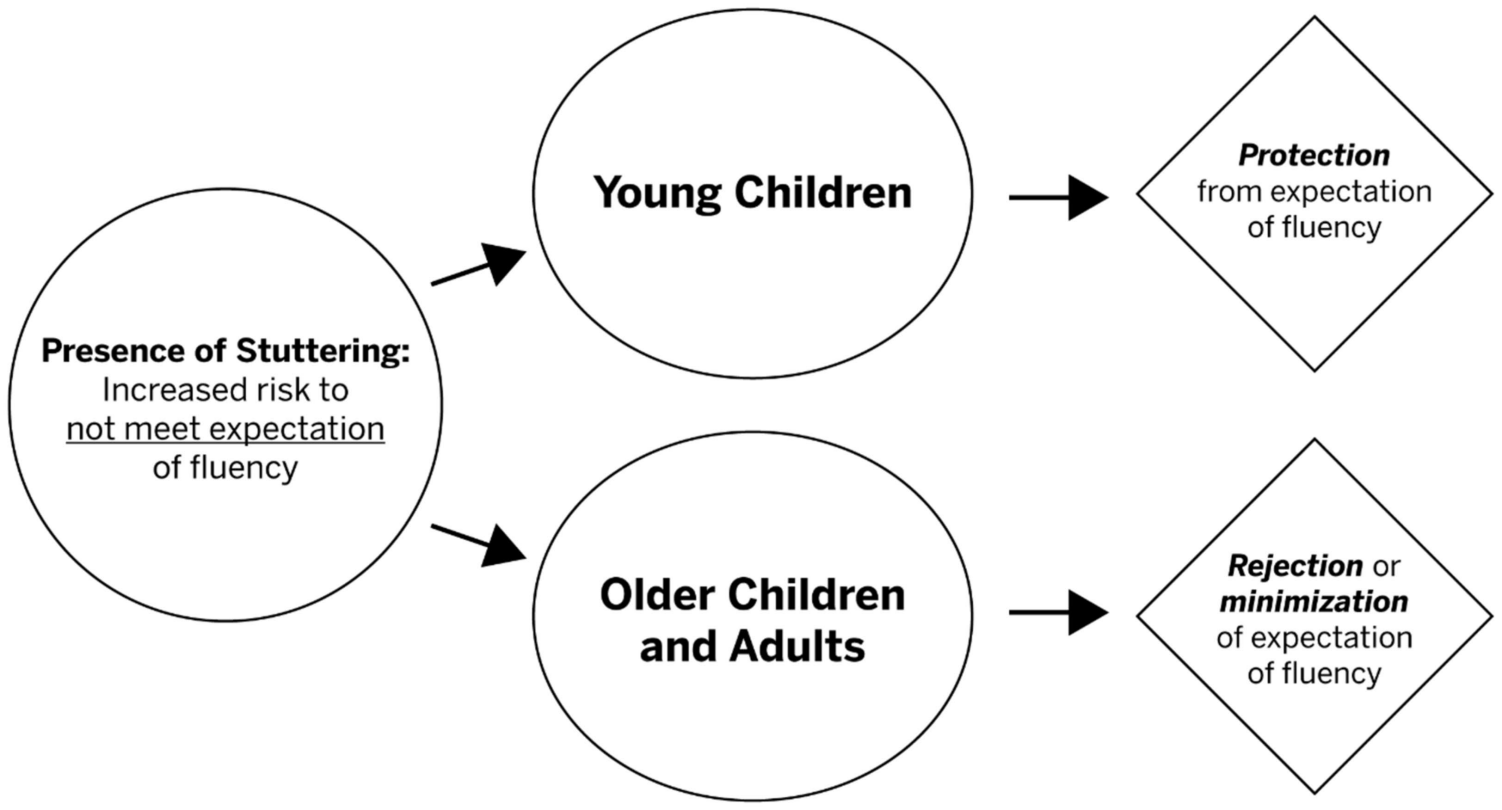
The CT model attempts to protect (young children) and reject/minimize (older children, adults) from the expectation of fluency. The CT assumes that protecting as well as minimizing an expectancy of fluency appreciably contributes to improvement in QOL.

Third, as shown in Figure 2, the CT model assumes that stuttering places individuals at greater risk of being unable to meet the expectation of fluent speech (far left side of Figure 2). Thus, the model attempts to prevent younger children from depending on such an expectation and to empower older children and adults to reject or minimize such internal and/or external expectations of fluency.

The latter point—being at risk of not meeting the expectation of fluency—appears to be related to an earlier theory (Sheehan, 1970) that depicting the risk of internalizing societal stigma regarding stuttering. Attempting to make such speculation less abstract, Sheehan employed a concrete object (i.e., an iceberg) to instantiate an abstraction (i.e., an internalization). Specifically, Sheehan concretized internalization by proposing the “iceberg of stuttering.” This metaphor assumes that those aspects of stuttering that are visible or “above the waterline” are smaller than the invisible or “below the waterline” aspects of stuttering. Some FT-related treatments for stuttering address this putative iceberg. We have no concerns with such attempts and instead focus our concern on how FT approaches to stuttering often involve an after-the-fact or reactive approach to the iceberg. In other words, these approaches would seem to be a reactive means to ameliorate the less visible or below-the-waterline aspects of the iceberg (i.e., the less publicly visible internalization of the stigma associated with stuttering) *after* it has begun to form. We believe this approach is problematic, particularly for children, because it seems to assume that they will inevitably form negative attitudes about their communication.

Additionally, the FT does not appear to consider whether its application may contribute to the development of a potentially deleterious, dependent relationship between fluency and QOL. In other words, we believe that FT may contribute, in whole or in part, to an internalized expectation of fluency that may adversely impact QOL. For example, if one is asked to try to stutter more easily and/or to make attempts to increase fluency, the implicit suggestion is that less stuttering is not only possible but a desirable outcome. In contrast, the CT posits that these goals contribute to the formation of a dependent and potentially harmful relationship.

Similar “camouflaging” techniques have been demonstrated to have long-term deleterious effects on the mental health of other stigmatized populations, including individuals with autism (e.g., Botha and Frost, 2018; Cassidy et al., 2020; Evans et al., 2024; Hull et al., 2017; Hull et al., 2021; Keating et al., 2024; Mantzalas et al., 2022; Perry et al., 2022; Ross et al., 2023; Zhuang et al., 2024) and those encouraged to code-switch in-and-out of Black African American Vernacular (Durkee and Gómez, 2022; Hudson et al., 2020; Johnson et al., 2021; McCluney et al., 2021; see Roberts, 2024, for review). Ethical concerns about the internalized stigma perpetuated by clinical goals designed to camouflage outward symptoms of autism (Ne'eman et al., 2023; Wilkenfeld and McCarthy, 2020) may also be relevant to the (in)direct fluency-focused goals of the FT. In other words, even when the fluency of their speech is not directly targeted, if reduction in stuttering is presented as a potential positive outcome, the perspective of less stuttering as a positive is reinforced. To be clear, it is not our intent to imply that autism and stuttering are the same, and/or that the lived experiences are comparable. The similarity across these groups, we propose, is the relentless societal pressure to conform to a normative communicative standard. The CT attempts to address these concerns by preventing young children from developing an internalized expectation of fluency in the first place.

Fourth, the CT assumes a future for children and adults within which the fluency of their speech is neither prohibitive nor indicative of communication effectiveness. Indeed, the CT assumes that stuttering openly is critical to communicating effectively and envisions a future in which their communication is no longer evaluated by themselves or others on the basis of the degree to which they do or do not stutter when speaking. The FT, however, appears to assume that a child’s future QOL is dependent on their ability to speak more fluently and that the inability to do so will lead to negative consequences. We believe that this perspective results in the development of a dependent relationship between fluency and QOL. The CT assumes that this dependency compromises communication, as efforts to speak fluently and/or stutter more easily directly conflict with efforts to stutter openly and to communicate effectively.

### 1.6 The CT model: some salient assumptions

Some salient assumptions related to the CT are described below.

Assumption 1: That communication effectiveness is a construct independent from changes in speech fluency and/or modification of stuttering.
Assumption 2: That therapeutic approaches directly addressing communication effectiveness from a wholly pragmatic perspective can significantly improve QOL.
Assumption 3: That therapeutic approaches that emphasize stuttering openly, and have no (in)direct goals designed to increase fluency or reduce or modify stuttered speech can yield positive changes in communication effectiveness.
Assumption 4: That older children and adults with considerable experience viewing themselves through the lens of stuttering stereotypes will benefit from therapy designed to reject or minimize this internalized expectancy of speaking fluently.
Assumption 5: That younger children can be prevented from viewing themselves through a lens of stereotypes of stuttering when their perception of their ability to communicate is not dependent on fluency.
Assumption 6: That gains in communication effectiveness and QOL are maximally obtained when supplemented by strengthening advocacy, resiliency, knowledge about stuttering and communication, and stuttering openly.

These assumptions and the four components of the CT are defined below.

## 2 Components of the CT model

### 2.1 Basic definitions and descriptions

The development of CT’s four components (i.e., Communication, Advocacy, Resiliency, Education) was based on the results of several years of applied, basic, and qualitative research (e.g., Byrd et al., 2018; Byrd et al., 2021; Byrd et al., 2022; Coalson et al., 2024). A definition and description of each of the four components is presented below, detailing how each of the components relates to treatment outcome and how each relates to the treatment’s endpoint: QOL.

#### 2.1.1 Communication

**Exchanging, imparting, or providing information, understanding, and support.** Within the CT, this component is considered the keystone that supports human connection. The CT is based, in part, on the belief that communication effectiveness, as indexed by perceived communication effectiveness by self and others, strengthens human connection and contributes to QOL.

The CT’s communication component presumes—based on a consensus among various theoretical models of communication—that communication effectiveness cannot be defined by the presence of a single feature (e.g., fluent speech production). For example, Spitzberg outlined more than 20 features of effective communication, only one of which was related to fluency (Spitzberg, 2013; Spitzberg and Cupach, 2011). Of particular relevance to the CT, Spitzberg’s model describes several other behaviors as contributing to communication effectiveness (e.g., eye contact, facial expression, speaking rate, organization of spoken content, listening, turn-taking, questions).

In attempts to amalgamate these various behavioral features contributing to communication effectiveness, Spitzberg (2007) developed measurable criteria to assist the National Communication Association in evaluating interpersonal communication skills. Spitzberg’s criteria, together with other criteria that center on those communication behaviors that are distinctly applicable to presentation format (Morreale et al., 2007), are foundational to CT’s communication component. Thus, these criteria inform the discrete pragmatics of verbal communication (e.g., vocal loudness, speaking rate, and emphasis) and nonverbal communication (e.g., body positioning, gestures, and facial affect) that are taught as well as evaluated with regard to the communication component of the CT.

Thus, this component of the CT attempts to help individuals enhance their communication effectiveness in academic, social, and vocational settings, with stuttering openly considered fundamental to communicating effectively. The model presumes that this enhancement of communication effectiveness counteracts the deleterious impact of attempts to fix or hide stuttering on communication, in addition to psychosocial and vocational health.

#### 2.1.2 Advocacy

**The apparent, demonstrable, and/or public support or recommendation of a particular cause, idea, policy, or point of view.** Within the CT, advocacy is thought to help support the development of empathy. In other words, the CT is based, in part, on the belief that self-advocacy helps educate others about stuttering within their immediate environment, and thus mitigates negative stereotypes and empowers the self.

The CT presumes that negative stereotypes regarding stuttering are pervasive. For example, stuttering is most commonly misperceived to indicate the individual is shy, nervous, weak, and unintelligent (see Craig et al., 2003); this has been well-documented among the general public (e.g., Bebout and Arthur, 1992; Boyle, 2017; Klassen, 2001), the media (e.g., Evans and Williams, 2015), across the lifespan (e.g., 3–5 years of age: Ezrati-Vinacour et al., 2001; 5–7-year-old: Giolas and Williams, 1958; 9–11 years of age: Franck et al., 2003; adolescents: Evans et al., 2008; adults: Van Borsel et al., 2011) and across a variety of professions (e.g., teachers: Dorsey and Guenther, 2000; Lass et al., 1994; protective service: Li et al., 2016; human resource/vocational counselors: Abou-Dahech and Gabel, 2020; Hurst and Cooper, 1983), including speech-language pathology (e.g., Anderson and Stuart, 2017; Cooper and Cooper, 1996; Woods and Williams, 1971). Indeed, self-advocacy is viewed as necessary to challenge societal stereotypes that characterize people with disabilities as being inferior (e.g., Keller and Galgay, 2010; Coalson et al., 2022).

The CT’s advocacy component presumes that participants developing elements of self-advocacy about their stuttering will contribute to the ability to inform other people about stuttering in a manner that challenges the stereotypes. Importantly, this development has been documented to improve self-perception as a communicator, their willingness to seek and persist through challenging communicative exchanges, as well as their overall self-confidence (e.g., Young et al., 2023). Thus, the advocacy component of the CT attempts to help speakers understand the value, both to themselves and their listeners, in sharing relevant issues regarding their stuttering (e.g., Byrd et al., 2017a; Byrd et al., 2017b; Croft and Byrd, 2021; Young et al., 2022; Young et al., 2023). The advocacy component is also assumed to enhance the ability to effectively advocate for themselves before, during, and after communicative exchanges.

#### 2.1.3 Resiliency

**A process that includes the ability to recover, adapt, or return to baseline following adversity.** Within the CT, resiliency is thought to be germane to an individual’s mental well-being. As such, the CT assumes that resilience serves, at least in part, as a buffer between (a) the external negative reactions to stuttering that a speaker may encounter and (b) the speaker’s internal (i.e., self) rating of their QOL.

The CT’s resilience component presumes, based on previous findings (e.g., Croft and Byrd, 2023; Freud and Amir, 2020; Winters and Byrd, 2021), that there is no significant correlation between lower levels of resilience traits and their stuttering frequency or severity. Self-compassion, a core component of resilience, is characterized by an open, caring, and nonjudgmental response to one’s own thoughts and feelings, especially in the face of negative experiences or emotions. In summary, findings of lower resilience-related traits, such as self-compassion (e.g., Croft and Byrd, 2020), relate to lower self-appraisal as a communicator (e.g., Croft and Byrd, 2023; Werle et al., 2021) independent of stuttering frequency and severity, even at young ages (e.g., Winters and Byrd, 2021).

Based on these findings, it would not be expected that QOL—a construct related to one’s resilience—will reliably improve by focusing clinical efforts on increasing fluency or modifying stuttered speech. In other words, improving one’s fluency or modifying one’s stuttering would not be expected to change, impact, or improve one’s resilience. Thus, the resilience component of the CT does not involve changes in fluency or stuttering. Rather, the CT attempts to strengthen resilience by helping individuals develop the ability to recover, adapt, or return to baseline in response to adverse communicative events if/or when they occur. The CT assumes that developing this ability will enhance resilience in the face of stuttering stigma. The desirability of this component of the CT is supported by the report of significantly lower resilience in adults (e.g., Freud and Amir, 2020).

#### 2.1.4 Education

**An enlightening experience that facilitates learning and future teaching.** Within the CT, education is considered fundamental to an individual’s empowerment. In essence, the CT rests, in part, on the belief that education involves increasing their knowledge of stuttering, communication, and misconceptions related to both. Strengthening this understanding is thought to help change or prevent the internalized stereotype of stuttering that many may develop. Research findings reveal that such stereotypes are reported by children early in life (Vanryckeghem et al., 2005) and adults (Boyle et al., 2023), as well as by their caregivers (Winters and Byrd, 2024).

The education component of the CT involves learning and strengthen understanding of basic facts and frequent misperceptions about stuttering. The information reviewed includes but is not limited to issues pertaining to the incidence and prevalence (Yairi and Ambrose, 2013), the variability of stuttering (Constantino et al., 2016), factors believed to contribute to the onset of stuttering [genetics: Frigerio-Domingues and Drayna, 2017; neurophysiology: Chang et al., 2019; cognitive/phonological factors: Ofoe et al., 2018; Ofoe et al., 2023; co-occurring conditions: Blood et al., 2003 (phonology), Druker et al., 2019 (attention deficit hyperactivity disorder); Elsherif et al., 2021 (dyslexia); Howell and Davis, 2011 (cluttering)], and just as importantly, factors that are misperceived as contributors to stuttering (e.g., multilingualism: Byrd, 2018; nervousness: MacKinnon et al., 2007). Similarly, the education component also fosters an understanding of communication competence and the skills they can strengthen to communicate effectively, further highlighting that stuttering and communication are independent constructs (e.g., Byrd et al., 2024a; Coalson et al., 2024; Coalson and Byrd, 2024).

The education component of the CT attempts to mitigate the well-documented, inaccurate beliefs about stuttering held by people of all ages (Craig et al., 2003; Ezrati-Vinacour et al., 2001) across a variety of vocational and professional settings (e.g., Abou-Dahech and Gabel, 2020), that perpetuate the pervasive stigmatization. Thus, the CT’s education component empowers children and adults to become effective advocates on behalf of themselves and the greater community as it relates to both stuttering and communication.

### 2.2 Empirical evidence

#### 2.2.1 Several paradigms

To date, we have employed several paradigms to empirically study the CT and its related assumptions. Some paradigms have been non-clinical or experimental in nature. For example, comparing the impact of communication effectiveness vs. speech fluency on listener perceptions. Other studies were more applied or clinical in nature, for example, evaluating pre-vs. post-treatment changes in CT components. Other studies have examined the changes in QOL before and after treatment. In passing, it is important to note that, to date—after seven publications on clinical studies of using the CT for children and adults—there have been no reported nor observed adverse effects associated with the model’s treatment approach.

For the purpose of this paper, most presented empirical studies of the CT involve group findings. Several of the measures associated with these studies consisted of self-ratings individuals who participated in the treatment (Byrd et al., 2016a,b, 2018, 2021, 2022; Coalson et al., 2024) as well as the ratings of relevant stakeholders (i.e., clinicians, caregivers, and/or untrained observers; Byrd et al., 2016a,b; Byrd et al., 2018; Byrd et al., 2024b). These measures were employed to evaluate each of the four CT components mentioned above. Reported sample size, *p* values, and effect sizes (ES) are provided or, if unreported, re-calculated based on extant data (if available).

#### 2.2.2 Social validation

The extent to which positive post-treatment effects are observed between groups is evaluated by social validation studies. Such studies provide subjective evaluations carried out by untrained individuals naïve to the purpose of treatment with no vested interest in the outcomes (see Schloss et al., 1987 for social validation in stuttering research). In general, the results of our empirical studies—whether basic or applied in nature—provide support for the notion that listeners do not necessarily prioritize fluency when assessing communication effectiveness.

For example, Werle and Byrd (2022a,b) had untrained observers (i.e., professors; *n =* 238, 158) rate the communication effectiveness of a videotaped speaker who produced 15% stuttering-like disfluencies, with findings indicating that communication is significantly higher when that speaker demonstrated stronger communication behaviors than when these behaviors were absent (*p* values: < 0.01, *d* values = 0.66 [medium ES], 2.15 [large ES]). Critically, relative to the underlying assumption of the CT, stuttering frequency and severity, and the content were identical between the two (i.e., high vs. low communication effectiveness) video samples.

More recently, Byrd et al. (2024a) had 81 untrained observers view one of two videos of an adult during a mock interview, either the one recorded 1 week before this adult participated in CT or the one recorded after their participation in CT. Although pre-and post-treatment samples were comparable in stuttering frequency and severity, post-treatment video samples were rated significantly higher in communication effectiveness than the pre-treatment samples [*p* < 0.001, *f^2^* = 0.36 (large ES)]. Within the same study, significant pre-to post-treatment gains in communication effectiveness were replicated [*p* = 0.045, *f^2^* = 0.24 (medium ES)] when a larger group of untrained observers (*n =* 96) viewed an adult with higher post-treatment frequency and severity of stuttering. Together, these studies provide social validation of the fundamental premise of the CT, that one can stutter openly and frequently and, with a strengthening of overall communication competence, be viewed by the public as an effective communicator. A third social validation study replicated these findings with a larger group of adult participants (*n =* 10) as well as a larger group of untrained observers [*n =* 1,110; *p* = 0.007, ηρ^2^ = 0.007 (medium ES)], and with the inclusion of two different speaking contexts (dyadic interviews, oral presentations; Coalson and Byrd, 2024). Although we have examined CT from the perspective of the untrained observer, we prioritize the speakers’ experience, not so that they can be more pleasing to the listener when they speak, but so that they can be maximally pleasing to themselves when they speak, which, because they stutter, would include times when they are stuttering, with no differentiation in their view of their communication depending on the fluency in their speech.

### 2.3 Children

#### 2.3.1 Non-clinical studies of CT components

##### 2.3.1.1 Communication

Winters and Byrd (2021) reported that in preschool-aged children (*n =* 59; age range: 2.5–6.9 years old), frequency, duration, and physical concomitants of stuttering did not significantly predict their self-rated communication attitudes. Similarly, in a larger cohort (*n =* 131; age range: 3.0–6.10 years old), Winters and Byrd (2024) found that stuttering severity scores did not moderate communication attitudes (*p* = 0.85). Together, these findings demonstrate that stuttering frequency is not the driver of their attitudes toward their communication abilities, lending further support to the CT’s rationale for focusing on communication through a pragmatic rather than a fluency lens.

##### 2.3.1.2 Advocacy

Byrd et al. (2016c) reported that after children self-disclosed their stuttering—a variable believed to be part of self-advocacy—listener-rated perceptions were significantly more positive, specifically in child listeners (*n =* 130, 6–12 years old, *p* = 0.013).

##### 2.3.1.3 Education

Non-clinical studies of education indicate that critical stakeholders in the lives of children hold outdated or stigmatizing attitudes about stuttering. For example, Byrd et al. (2020) reported that speech-language pathologists (SLPs; *n =* 141) were significantly less comfortable using the word “stuttering” in front of parents of children diagnosed with stuttering compared to three other common childhood diagnoses (articulation, language, and phonological disorders, *p* < 0.001). Relatedly, Winters and Byrd (2020) reported that pediatricians (*n =* 122) were significantly less likely to provide referrals for children who demonstrate fewer overt stuttering symptoms [*p* < 0.0001; ηρ^2^ = 0.52 (large ES)] compared to children with more overt stuttering behaviors.

###### 2.3.1.3.1 Resiliency

To date, non-clinical data on resilience have been collected and are currently being processed for peer review.

##### 2.3.1.4 QOL

Studies investigating measures of QOL in children have been restricted to clinical studies (Section 2.3.2.3) and are further described in published non-clinical (Section 2.4.1.5) and clinical studies (Section 2.4.2.4) with adults.

#### 2.3.2 Clinical studies of CT components

##### 2.3.2.1 Communication

Byrd et al. (2021) investigated pre-CT vs. post-CT changes in the communication competencies of children (*n =* 37). Based on the ratings of a speech-language pathologist (*n =* 1) not associated with their treatment, there were significant pre-to post-treatment increases in eight of the nine core communication competencies targeted by CT [*p*-value range: < 0.001 to <0.005; *d* value range: 0.01–1.30 (small to large ES)] (e.g., speaking rate, vocal loudness, language organization, eye contact, etc.). Byrd et al. (2024b) replicated this finding in a larger cohort of children (*n =* 61) and found that pre-to post-treatment gains were observed for seven of the nine core communication competencies [*p* value range: < 0.01 to <0.02; *d* value range: 0.32–1.41 (medium to large ES)].

##### 2.3.2.2 Resiliency

A variable closely related to resiliency is the establishment of positive peer relationships (e.g., Graber et al., 2016; Noble and McGrath, 2011). Results of three investigations [Byrd et al., 2016b (*n =* 23); Byrd et al., 2021 (*n =* 37); Byrd et al., 2024b (*n =* 61)] indicated that there were significant pre-to post-CT increases in self-or caregiver-reported perceived ability to establish peer relationships post-treatment [*p* value range: <0.001 to <0.005; *d* value range: 0.27–0.61 (small to medium–large)]. These self-reports were measured using the Patient Reported Outcomes Measurement Information System (PROMIS) Pediatric Peer Relationships Form (DeWalt et al., 2013).

###### 2.3.2.2.1 Advocacy and Education

To date, no clinical studies focusing on post-treatment changes in Advocacy or Education components of the CT have been published for children. The authors have collected and are currently processing these data.

##### 2.3.2.3 QOL

Over the last decade, refinements in CT and the impact it has on the QOL have been assessed as a collective, without particular focus on the four different CT components. Across these early studies, changes have been observed in the adverse impact of stuttering and the perceived ability to make friends (e.g., Byrd et al., 2024b, 2016a, 2016b, 2018, 2021). More recent investigations have been conducted to determine the relative contribution of each of the four components to the outcomes observed.

Prior studies of children have included the Overall Assessment of Speaker Experience of Stuttering (OASES; Yaruss, 2010; Yaruss and Quesal, 2016) as a pre-/post-treatment outcome measure, which includes a measure of QOL (OASES-Section 4). Byrd et al. (2016a) found significantly higher post-treatment ratings on this measure compared to pre-treatment (*n =* 23, *p* = 0.013). This was replicated by Byrd et al. (2018) in a different cohort of children (*n =* 23) using the same OASES measure [*p* = 0.004; *d =* 0.67 (medium–large ES)]. Non-significant post-treatment increases in QOL were reported by Byrd et al. (2021) (*n =* 37, *p* = 0.40). In the largest cohort to date (*n =* 61), Byrd et al. (2024b) reported significant gains in QOL following CT [*p* < 0.01, *d =* 0.52 (medium ES)].

### 2.4 Adults

#### 2.4.1 Non-clinical studies of CT components

##### 2.4.1.1 Communication

Werle et al. (2021) reported that adults self-rated their communication effectiveness significantly lower than their ratings of adults who do not stutter [*p* < 0.05, *d =* 0.59 (medium ES)]. In addition, Werle and Byrd (2022a,b) reported that untrained observers (*n =* 238, *n =* 158) rated a presenter demonstrating high communication effectiveness and 15% stuttering frequency significantly higher than a presenter with 0% stuttering and low communication competence [*p* values: <0.01; *d* values = 0.66 (medium ES), 2.15 (large ES)]. Werle et al. (2023) also found that listeners reported stuttering to be significantly less distracting when observing a presenter with high communication effectiveness vs. the same presenter with low communication effectiveness [*p* < 0.01; *d =* 0.83 (large ES)]. Notably, in both cases, the presenter exhibited identical stuttering frequency and severity. These studies, in addition to the replicated findings of our social validation studies (Byrd et al., 2024a; Coalson and Byrd, 2024), provide evidence corroborating the distinction between stuttering and communication competence from the perspectives of both the speaker and untrained observers.

##### 2.4.1.2 Advocacy

Qualitative data collected by Coalson et al. (2022) from a focus group of adults (*n =* 7) found that they may exonerate the listener for microaggressive comments or actions about their stuttering and, at times, even experience feelings of guilt that persons they engage with have to listen to their stuttering—guilt that often makes them feel that they should apologize for the way they talk. Byrd et al. (2017a,b), however, reported that listeners rated their perceptions of adults significantly higher after they disclosed to them that they stutter in a non-apologetic manner, as compared to an apologetic self-disclosure of stuttering [*p* values: < 0.0001 to 0.0004; ηρ^2^ values: 0.05–0.08 (medium ES)]. Byrd et al. (2017b) further reported no significant difference in listener perceptions of adults when they provided an apologetic disclosure of stuttering than when they provided no disclosure.

Croft and Byrd (2021) subsequently found that listeners rated adults significantly higher in personality traits after non-apologetic self-disclosure of stuttering [*p* = 0.004, ηρ^2^ = 0.12 (large ES)]. Similarly, Werle and Byrd (2022b) reported that listener ratings were significantly higher for a presenter who self-disclosed and exhibited 15% stuttering than for the same presenter without disclosure and 15% stuttering. Likewise, listeners were significantly less distracted by a presenter with high communication effectiveness, self-disclosure, and 15% stuttering than the same presenter, and presentation, with no disclosure and 15% stuttering (Werle et al., 2023).

Young et al. (2022) conducted a phenomenological analysis of interviews with 12 adults and found that these individuals describe non-apologetic self-disclosure as an effective means to achieve greater cognitive relief, self-empowerment, and social connection. In a subsequent, larger, mixed-model study, Young et al. (2023) (*n =* 156) reported that, similar to the adults in the 2022 study, self-disclosure was described as beneficial for almost all participants (96%), with significant benefits for confidence [*p* < 0.02; Cramer’s *V* = 0.242 (small-medium ES)] and reducing avoidance [*p* < 0.001; Cramer’s *V* = 0.334 (medium ES)]. Interestingly, an inferential analysis did not detect a significant impact of self-disclosure on participants’ immediate or long-term speech fluency (*p* = 0.461), lending additional support to the CT assumption that these positive changes occur independently of changes in stuttered speech.

##### 2.4.1.3 Resiliency

Croft and Byrd (2020) (*n =* 70) reported a significant relationship between greater adverse impacts of stuttering and lesser self-compassion [*p* < 0.0001, *R^2^* = 0.43 (large ES)] with self-compassion defined as self-kindness, mindfulness, and social connectedness. This finding is germane to the CT’s attempts to foster resilience because self-compassion is a critical factor in one’s ability to be resilient following challenging experiences (Neff, 2023, see also Chuang et al., 2023; Deniz et al., 2022; Ewert et al., 2021). Croft and Byrd (2023) (*n =* 96) reported that resilience (as measured by self-compassion) in adults significantly predicted decreased rumination [*p* = 0.009; *R^2^* = 0.04 (medium ES)] about perceived communicative effectiveness during oral presentations. Further, greater resilience was not significantly associated with observer-rated stuttering severity (*p >* 0.05).

One primary objective of CT’s aim to foster resilience is engaging in the act of voluntary stuttering. Voluntary stuttering can be considered a challenging activity, likely due to the connection to internalized stigma. However, to challenge oneself to engage with moments of stuttering in an authentic manner (rather than “easy stuttering”) and then return to these moments with greater self-compassion and positive self-perception is critical to strengthening resilience. Byrd et al. (2016d) conducted the largest study investigating the benefits of voluntary stuttering (*n =* 206), finding that adults reported significant gains in QOL after using voluntary stuttering, provided that it was (a) similar to their actual moments of stuttering [*p* < 0.001; Cramer’s *V* = 0.247 (medium ES)] and (b) used more than once beyond the clinic [*p* < 0.001; Cramer’s *V* = 0.510 (large ES)].

##### 2.4.1.4 Education

A thematic analysis by Young et al. (2022) found that adults (*n =* 12) report a positive impact on their listeners of informative self-disclosure (e.g., “I stutter, so you may hear me repeat sounds or words”). Young et al. (2023) found that adults (*n =* 156) consider informative disclosures of stuttering to have a significant, positive effect on their own confidence during speech production [*p* < 0.02; Cramer’s *V* = 0.242 (small–medium ES)] and lower levels of avoidance [*p* < 0.001; Cramer’s *V* = 0.334 (medium ES)]. These data indirectly support the role of education, as without an understanding of stuttering, it is difficult to effectively provide an informative statement.

##### 2.4.1.5 QOL

Non-clinical studies investigating measures of QOL with respect to CT components include both phenomenological data (i.e., interviews by Young et al., 2022, 2023) and quantitative data (as measured by OASES-Section 4; Croft and Byrd, 2020).

###### 2.4.1.5.1 Advocacy

As described above, Young et al. (2022) conducted a phenomenological (non-parametric) study that thematically analyzed the self-disclosure (a variable related to self-advocacy) of adults (*n =* 12). Results indicated that adults found self-disclosure of stuttering as a factor contributing to (a) cognitive relief, (b) self-empowerment, and (c) social connection. The participants described these contributions as improving their QOL. A larger mixed-methods study (*n =* 156) by Young et al. (2023) found that 96% of adults described self-disclosure of stuttering, particularly non-apologetic disclosure, as beneficial for their confidence as a communicator.

###### 2.4.1.5.2 Resiliency

Croft and Byrd (2020) studied self-compassion (a variable related to resilience) reported by adults (*n =* 140). In this non-clinical study, these researchers reported that increased self-compassion was significantly correlated [*r* = 0.626 (large ES); *p* = 0.007] with their increased QOL (as measured by OASES, Section 4). In a similar study, Croft and Byrd (2023) (*n =* 96) reported that increased rumination was significantly correlated [*r* = 0.52 (large ES); *p* < 0.001] with decreased QOL (as measured by the Total OASES Score).

###### 2.4.1.5.3 Communication and Education

To date, empirical, non-clinical studies of adults have not been conducted but are planned for the future.

#### 2.4.2 Clinical studies of CT components

##### 2.4.2.1 Communication

Byrd et al. (2022) (*n =* 11) reported a significant pre-to post-treatment increase in clinician ratings of communication effectiveness in eight of the nine core communication competencies [*p* value range: < 0.01–0.02; *d* value range: 0.83–1.41 (large to large ES)]. The relatively small number of adults who participated in this study also reported that their self-rated communication effectiveness non-significantly increased from pre-to post-CT [as measured by the Self-Perceived Communication Competence scale (McCroskey and McCroskey, 1988)]. However, recently, Coalson et al. (2024), employing a larger sample of adults (*n =* 33), reported a statistically significant [*p* < 0.001; *d =* 0.70 (medium–large ES)] pre-to post-treatment increase in self-ratings of communication effectiveness using the same measure.

##### 2.4.2.2 Resiliency

Byrd et al. (2022) reported significant post-CT gains in resilience for adults [*n =* 10; *p* < 0.001, *d =* 1.13 (large ES)], as measured by the Devereux Adult Resilience Survey (MacKrain, 2008). Similarly, the same authors reported positive (yet non-significant: *p* = 0.12) post-treatment gains in self-compassion (as measured by the Self-Compassion Scale; Neff, 2003). Clearly, further pre-vs. post-treatment studies of resilience —relative to the FT—are warranted; however, the above preliminary findings were taken to suggest that CT is associated with improvements in elements of resilience.

###### 2.4.2.2.1 Advocacy and Education

To date, clinical studies of adults have been conducted and are currently being prepared for peer review.

##### 2.4.2.3 QOL

Using the OASES Section 4 as a measure of QOL, Byrd et al. (2022) reported significant gains post-treatment for adults [*n =* 11, *p* < 0.01; *d =* 1.01 (large ES)]. In a subsequent, larger study (*n =* 33), Coalson et al. (2024) reported significant gains following CT [*p* < 0.001; *d =* 1.24 (large ES)] based on Total OASES Score. Total OASES Score is considered to reflect the speaker’s positive or negative experiences with stuttering. Although significant gains were detected for Section 4 (QOL), these findings were underpowered and, therefore, published findings were conservatively restricted to the more adequately powered Total OASES Score.

## 3 Discussion

### 3.1 Findings to date regarding CT: an overview

#### 3.1.1 Non-clinical studies of children

Our preliminary non-clinical findings indicate that the frequency, duration, and physical concomitants of stuttering do not significantly predict the self-rated communication attitudes of preschool-aged children. This finding is consistent with CT’s basic assumptions and tenets. Regarding non-clinical studies of advocacy, findings are taken to suggest that children who self-disclose their stuttering are attributed more positive personality traits by their peers than when they do not disclose. In terms of education about stuttering, both SLPs and pediatricians, as a group, reported less-than-informed approaches to conversing with and/or referral for stuttering treatment for children. For instance, the authors reported that SLPs are reluctant to use the word stuttering in the presence of parents and their children. Such reluctance supports the notion that stuttering and/or its mention are associated with negative connotations and outdated stereotypes.

Overall, preliminary findings from non-clinical studies of children regarding three of the four components of CT appear to be consistent with the underlying tenets of the model (communication, advocacy, and education). Additional non-clinical data focusing on resiliency in children have been collected and are under preliminary review for dissemination in a scholarly journal.

#### 3.1.2 Clinical studies of children

The authors reported preliminary findings from clinical studies indicating that communication in school-age children significantly increased from pre-to post-CT. Further, various indexes of stuttering do not appear to predict changes in their communication effectiveness. In terms of resilience, preliminary findings indicate that their ability to form new peer relationships is rated significantly higher following CT. We are currently conducting clinical studies of the other two CT model components—advocacy and education—as well as on how changes in all four components relate to QOL for children.

In summary, and of particular salience to the CT, the preliminary findings from clinical studies of children indicate that significantly increased pre-to post-CT communication effectiveness does not predict post-CT changes in various indexes of stuttering. In other words, children’s communication effectiveness can significantly improve without commensurate improvement in stuttering.

#### 3.1.3 Non-clinical studies of adults

Similar to our findings regarding children and communication effectiveness, preliminary non-clinical findings indicate that adults’ self-perceived communication effectiveness is often unrelated to stuttering frequency or severity. These results are corroborated by a recent social validation study that suggests a similar dissociation. The authors have taken these findings to suggest that communication plays an important role in listener perceptions, even when the adult speaker is stuttering to a higher degree than they were prior to participating in CT.

Regarding variables associated with advocacy, preliminary findings suggest that self-advocacy in the form of non-apologetic self-disclosure of stuttering has a positive impact on listeners’ perception of adult speakers. This self-disclosure was also found to have a positive impact on the speaker’s own communication experience, with adults reporting that they could focus more on their communication rather than on whether or not they stuttered. Likewise, when the stuttering frequency was held constant (i.e., 15% stuttering frequency) for two different presentations, listeners rated presentations following self-disclosure for the same adult as less distracting when that individual presented with high vs. low communication effectiveness. Similar positive benefits of advocating for oneself via self-disclosure of stuttering were reported in both qualitative and large-scale studies of adults.

Results on variables related to the resilience of adults suggest that self-compassion (a variable associated with resilience) plays a critical role in the impact of stuttering on the speaker. Specifically, when adults exercised self-compassion, it mediated the degree of rumination about their stuttering specific to their communicative performance.

Regarding education, preliminary findings regarding the positive impact of providing information to the listener during self-disclosure (i.e., informative self-disclosure of stuttering) appear to be consistent with the underlying assumptions of the CT. Together, these findings from non-clinical studies with adults on variables associated with communication, advocacy, resiliency, and education, though preliminary in nature, appear to be consistent with the underlying tenets of the CT.

#### 3.1.4 Clinical studies of adults

With regard to clinical findings, we have published research demonstrating that adults exhibit pre-to post-CT increases in communication effectiveness, a finding based on both clinician-as well as adult participants’ self-ratings. Similarly, there are preliminary data indicating significant pre-to post-CT increases in their resilience. We acknowledge that these data are preliminary in nature and that further pre-vs. post-CT studies of resilience in adults are warranted.

#### 3.1.5 Summary of studies of children and adults

##### 3.1.5.1 Blank Center CARE™ Model of Treatment

Overall, while the results of these preliminary clinical studies with both adults and children participants are promising, further empirical studies with larger sample sizes, refined methods, measures, etc., are needed. This is particularly true for children, where there is less evidence regarding the relationship between QOL and CT outcomes.

##### 3.1.5.2 QOL

Regarding QOL, the authors’ preliminary findings suggest simultaneous changes in CT components (e.g., communication, advocacy, etc.) and changes in overall QOL reported by children and adults. Although again preliminary, we believe these findings support the notion that CT improves QOL. Having said that, we acknowledge that these results require additional replication and investigation in future studies with larger cohorts and modified methodology.

#### 3.1.6 Social validation

Social validation studies are used to evaluate the extent to which positive post-treatment effects are also observed by the general public. These studies involve subjective evaluations provided by untrained individuals naïve to the purpose of treatment who have no vested interest in the outcomes (see Schloss et al., 1987 for social validation in stuttering research). In general, the results of our empirical studies—whether basic or applied in nature—support the notion that listeners do not necessarily prioritize fluency when assessing the communication effectiveness. In particular, the previously discussed clinical studies by Byrd et al. (2024a) and Coalson and Byrd (2024) directly supports this supposition, as the general public viewed the speakers as effective communicators, even when the speaker stuttered more post-treatment. Further research is warranted, but these data provide burgeoning social validation for the fundamental premise of the CT, that when communication competence is strengthened, one can stutter openly and frequently and be viewed as an effective communicator.

### 3.2 Conceptual considerations

#### 3.2.1 Change in the zeitgeist

In general, we believe that the aforementioned study provides the motivating rationale for changing the zeitgeist in relation to stuttering treatment. This change or shift would necessitate a different conceptualization of such treatment, one that does not singularly focus on fluency shaping or stuttering modification. This new conceptualization is engendered, at least in part, by the notion that much of the present focus of stuttering treatment appears to be largely rooted in ableism (i.e., an approach that attempts to correct, cure, or fix a disabling condition).

We advocate for a possible shift in thinking away from trying to “fix or cure” stuttering (including attempts to make stuttering easier) to an approach that addresses whole-person wellness, wherein reducing stuttering to any degree is neither a direct nor an indirect target of treatment. Certainly, we understand that our thoughts on such a shift may not be shared by all. Disagreement with and/or reluctance to accept and adopt that which is different is natural. As Charles Kettering (1959) said, “The world hates change, yet it is the only thing that has brought progress.” If such a change does occur, it is likely to be incremental rather than quantal in nature. Such gradual transformation reflects an appropriately cautious and thoughtful means by which one changes their approach to, consideration of, and thinking on an issue.

Whether incremental or quantal in nature, we suggest the need to change the considerations and conceptualization of stuttering treatment: A move away from a singular or (in)direct focus on fluency via fluency shaping and modification of stuttering and a shift toward strengthening the overall pragmatics of the communication. This shift, we contend (with supporting data), should appreciably enhance the communication effectiveness as well as supporting variables (e.g., resiliency, advocacy). In doing so, we should materially contribute to the improvement, maintenance, and protection of the QOL across academic, home, social, and work settings.

#### 3.2.2 Change in paradigm

Of course, whether a shift from a more fluency-focused to a communication-focused paradigm will occur, in whole or in part, is currently unknown. Obviously, we believe such a shift should occur and would be desirable. Our belief is consistent with our previously discussed conceptualizations of how and why the treatment of stuttering warrants critical appraisal, evaluation, and modification. Further, this belief is also supported by the preliminary findings from both non-clinical and clinical studies of child and adult participants presented above. Certainly, the relative desirability, need for, and wisdom of such a shift will be a matter for continued debate, discussion, practice, and study. As part of this discussion, there is likely to be a concomitant change in how communication itself is conceptualized.

For example, adhering to a conceptualization of communication that importantly, solely, or even partly relies on fluency is likely to be challenging. This challenge arises from at least two facts: First, communication effectiveness relies on more—much more—than speech fluency. It involves a host of variables (i.e., Spitzberg, 2013; Spitzberg and Cupach, 2011), of which speech fluency is only one. Simply put, the shoulders of fluency are not broad enough to support communication effectiveness on their own. Second, given the findings presented above—whether those related to children or adults—it appears quite possible that improvement in communication can occur with little or no change in stuttering frequency or severity. Third, some argue that CT has already been employed as a method of treatment for some time, yet as explicated throughout this paper, if there is any focus on modifying stuttering—direct or indirect to any degree—then that focus runs counter to the fundamental premise of CT. Fourth, learning to stutter more easily and decreasing avoidance of stuttering is often presented as the more positive path as compared to paths focusing on increasing fluency shaping. However, either way you slice it, both suggest less stuttering is possible and preferable.

As mentioned above, both non-clinical and clinical findings related to the CT model strongly support the notion that listeners do not seem to prioritize stuttering frequency or severity when judging the communication effectiveness. Having said that, as is also mentioned above, others (e.g., Kohmäscher et al., 2023; Menzies et al., 2008) have reported benefits for communication associated with stuttering modification or more traditional fluency therapy when combined with cognitive behavioral treatment. Nevertheless, our findings—from the perspective of children and adults as well as clinicians, and untrained observers—are quite clear on one point: changes in fluency are not necessary for significant changes in the communication effectiveness and QOL. As noted by a participant in the study of Young et al. (2022): “… I’m not focusing on getting out of it [stuttering moments], or changing it, or speaking as fast as I can to get through it. I’m focusing, I’m now reacting to what I’m saying and who I’m speaking to and how broad conversation is going. That’s definitely provided a sense of enjoyment. I am more of an extrovert, so I enjoy having conversations (p. 2050).”

Such qualitative data provide further evidence of the power of participatory research during clinical and empirical decision-making processes (Bourke, 2009; Fletcher-Watson et al., 2021; Gourdon-Kanhukamwe et al., 2023). In this regard, the development of the CT has been driven by participatory research, with the authors listening to the lived experiences of the children, adults, and families they have served for more than 20 years. These first-hand perspectives, as reflected in the CT, informed the need for a shift away from fluency as a primary, necessary, or desirable direct or indirect goal. Additionally, besides our research, other data have demonstrated a need for this paradigm shift. For example, of the 71 adults who responded to a survey request at the 1999 National Stuttering Association (NSA) conference, 42% reported that they benefited from “learning new speaking patterns that reduced or eliminated stuttered speech” and 61% benefited from “learning techniques to control their stuttered speech” (Yaruss et al., 2002). Ten years later, only 11% rated techniques to eliminate stuttering as “very successful” and 19% reported techniques to stutter “more easily” as “very successful” [1,235 NSA members (164 of which were SLPs); Executive Report of National Stuttering Association, 2009]. More recently, in 2021, a panel of 45 stakeholders surveyed with an e-Delphi Survey (13 of which were SLPs) ranked the statement “Working directly on speech to reduce the amount or severity of stuttering” 63rd in level of importance during clinical goal-setting (of 89 total statements; elimination of stuttering was not ranked; Connery et al., 2022). Statements related to communication, advocacy, resiliency, and education, however, were ranked higher in priority. CT therefore aligns with this feedback provided via lived experiences with stuttering and we will continue to listen to the voices of children adults and their loved ones as we explore the efficacy of this approach.

### 3.3 Communication and stuttering: The impact of FT

As noted above, FT appears to assume that therapeutic approaches such as fluency shaping and stuttering modification contribute to both enhanced communication effectiveness and QOL. One may argue, of course, that FT is not mainly or primarily associated with such an assumption. Rather, perhaps one of the main underlying assumptions for any FT is that it attempts to change, cure, or fix a disabling condition. There is nothing wrong with such a rationale if a change in fluency is the central or main *raison d’être* for the FT.

However, if the FT also assumes to significantly enhance, improve, or increase communication effectiveness by reducing stuttering, we would question this assumption—that increases in fluency increase communication effectiveness. We believe it is fair to ask, “where are the data to support such an assumption?” While some extant findings do appear to support this (e.g., Baxter et al., 2016), it seems reasonable to suggest that the extant literature is not replete with nonclinical or clinical research supporting FT’s impact on communication effectiveness. Clearly, more empirical work is needed—involving clinical, descriptive, and experimental studies—to better understand the relation between the FT and communication effectiveness. Such research, it might be added, should include, wherever possible, the perspective of (1) children and adults, (2) clinicians, and (3) untrained observers, in addition to the perspectives of the researchers conducting the study.

### 3.4 Communication and stuttering: The impact of CT

As suggested in Figure 2, CT differs from FT in the way it treats the expectation of fluency in young children. The FT approach seems to assume that children should expect to be fluent. In contrast, the CT approach focuses on the expectancy of enhanced communication effectiveness without regard to fluency. In essence, this CT approach, we would argue, helps protect the young child from internalizing the expectancy of fluency, an internalization that becomes problematic when fluency is not achieved. The CT explicitly attempts to help older children and adults to reject or minimize the expectancy of fluency to the highest degree possible. Thus, the CT aims to help older children and adults focus on enhancing their communication effectiveness rather than on increasing the fluency of their speech and/or attempting to stutter more easily as they are communicating.

Some approaches may continue to focus on acceptance of stuttering while simultaneously offering commentary and strategies that shift the focus to changing stuttering and—implicitly or explicitly, intentionally or unintentionally—perpetuating a preference for fluent speech (e.g., Van Riper, 1973; Yaruss et al., 2012). To be clear we recognize, as is noted in our discussion regarding voluntary stuttering, that learning about and lessening reactivity to individual moments of stuttering is invaluable to proactively preventing or ameliorating the potential distraction of stuttered speech. Thus, we agree with Van Riper in his remarkable early contribution to the field that stuttering modification and the related desensitization would strengthen communication. However, as explicitly stated by Van Riper, the intent of stuttering modification was also to reduce moments of stuttering. Specifically, in addition to acceptance through desensitization, Van Riper (1973) presented his hierarchical, stepwise stuttering modification approach, based on his anecdotal data that aimed to make the individual sound “normal”:

*Rejecting his old, abnormal preformations and tendencies to use hard contacts and sudden surges of tension, [the stutterer] plans to begin the feared word in a more normal fashion, integrating the timing of airflow and phonation and working slowly through the motoric sequence… It is a new, more adaptive behavior, a replacement that becomes condition to the antecedent conditions. … It is also one that transfers very easily into normal speech. The stutterer who stutters in this way can be very fluent (p. 338).*

We posit that this sends a conflicting message. First, that normal speech is defined by stuttering less and is preferred, and that increased fluency is the natural byproduct of desensitization. Our research indicates that, for some individuals who are particularly adept at hiding their stuttering, the process of getting comfortable with their natural way of talking actually yields greater, rather than lesser stuttering post-CT (see Coalson et al., 2024, p. 1976). Further, acceptance of stuttering is a critical step for some, but not all individuals, and one can accept that they stutter, but still avoid engaging in daily life. Thus, a fundamental premise of CT is that acceptance should not be needed, if rejection has yet to take place, and that treatment that targets reduction in stuttering may be unintentionally contributing to early and ongoing rejection, and compromise QOL by suggesting that it is ok to stutter but still engaging in dialogue that celebrates times when speech is more fluent and/or strategies with the aim of reducing stuttering.

These contradictions (i.e., stating that it is acceptable to stutter while appearing to either implicitly or explicitly focus on/prefer fluency) may make it difficult for an adult or child to disentangle stuttering from their perspective of themselves as communicators. For example, clinicians may reassure the children and adults they provide treatment to that it is ok to stutter but then advise them to try to stutter a little more easily [e.g., “…the literature contains numerous examples of comprehensive treatment approaches that address acceptance in addition to [rather than instead of] increased fluency…” (Yaruss et al., 2012, p. 537)]. Clinicians may also reassure the individuals they serve that it is important to fully embrace stuttering and to adopt the framework that “it’s ok to stutter,” but then make evaluative comments such as, “Did you notice when you just read that passage, you did not stutter? Way to go!” Or, they may reference the individual’s stuttering frequency as a better or worse day based on whether the frequency is higher or lower. For example, “Today seemed like a much better day than last week; Did you notice how fluent you were?!” Such mixed messages and (in)direct encouragement of camouflaging, as noted, have harmed other stigmatized populations (e.g., Botha and Frost, 2018; Cage et al., 2018; Cassidy et al., 2020; Evans et al., 2024; Hull et al., 2021; Keating et al., 2024; Mantzalas et al., 2022; Perry et al., 2022; Ross et al., 2023; Zhuang et al., 2024; also see Durkee and Gómez, 2022; Hudson et al., 2020; Johnson et al., 2021; McCluney et al., 2021; for further review, see Roberts, 2024).

One might analogize this contradiction to handedness. For example, such contradictions between “it’s ok to stutter, but let us still try to do it less” would be akin to saying “… it is ok to be left-handed, but let us try to write a little more with your right hand,” or “Sure it is ok to be left-handed, but it was great that you just wrote that whole sentence with your right hand, good job!” Children and adults infer from these exchanges that while it may be acceptable to stutter, it is not preferred, and consequently, they may exert more effort to avoid stuttering than the clinician ever intended. Such efforts, one might suggest, may temporarily increase fluency. However, within the framework of the CT, such increases are believed to come at the expense of communication effectiveness and QOL, and at the development and strengthening of a dependent relationship of their perception of their communication and how much they do or do not stutter. Of course, some clinicians may argue that if the individual requests fluency, we should provide that to them or at least attempt to. In response, we believe that the question remains, at what expense? And in doing so, what would the individual or clinician expect? Improved communication? The manner in which these aspects are operationally defined and supported in the literature warrant further consideration. Importantly, there is now an alternative to FT, one for which a fundamental premise is that any focus on fluency will compromise communication competence.

Despite that premise, the argument may be that if the individual asks for fluency, we should provide it. Yet the same argument does not seem to be asked of FT. There are a large number of programs that offer fluency, and somewhere along the way it has been accepted that these programs are targeting communication because they are attempting to help people to speak more fluently. We are not forcing any individual to forgo their efforts or desire to engage in any form of treatment for which the goals are to stutter less. Rather, we are positing that an individual seeking the treatment now has another choice, an alternative to FT—one that we believe is long overdue, and again, one that will be compromised in its effect if we are also attempting to increase fluency. Thus, if an individual participant seeks increased fluency as part and/or the main aim of treatment, they can seek clinicians who are specialized in providing that approach. However, if they are seeking an approach that will provide a path to communicating more effectively, in the manner communication is defined and evaluated throughout this paper, they can seek clinicians who are specialized in providing CT.

We further posit that our development of an approach for stuttering for which decreasing stuttering is not (in)directly targeted in and of itself is stigma reducing. Interestingly, historically, being left-handed was stigmatized. Many tried various techniques, some painful, to force the use of the right hand, with significant stress on their fine motor system, and, notably, their confidence in their ability to write like everyone else (e.g., Morsh, 1930; Hildreth, 1950; see Cornel, 2019 for review).^4^ The public perception of being left-handed resulted in a significant proportion of the population hiding their handedness. We are not suggesting that the lived experience of being left-handed is equivalent to the lived experience of stuttering, but the similarities do provide insight into how public forcing of conformity can shift to inclusivity with education over time.

In contrast to assuming that improvements in fluency are associated with the enhancement of communication effectiveness, the CT makes the basic assumption that improvement in communication need not be tied to and/or result from changes in speech fluency. Further, the CT takes into account that, in essence, requiring and or implicitly/indirectly suggesting that a person, whether a child, teen, or adult, to focus concurrently on both communication and increasing fluency, and/or modify moments of stuttering would seem quite challenging. This concurrent focus on communication and fluency is particularly concerning, we would argue, when the individual interacts outside of a supportive therapeutic environment. In such an environment, achieving this concurrent focus would be quite difficult, if not nearly impossible, as has been documented and previously discussed. We believe that this practice—attempting to have the speaker concurrently attend to or focus on both communication and fluency—may, in effect, turn that person’s speech (inside and outside of therapy) into a dual-attention task (e.g., using a mobile phone while driving a car). Such an apparent dual-attention approach, we suggest, may have deleterious impact on communication, fluency, or both.

### 3.5 Decoupling communication from fluency

The preliminary results of empirical studies of the CT provide reasonably strong initial support for the notion that changes in communication effectiveness in children and adults are independent of changes in stuttering. Further, as mentioned above, our preliminary findings support the contention that changes in fluency/stuttering are not necessary for positive changes in communication effectiveness. These findings support our suggestion that the decoupling of communication from fluency/stuttering has at least two benefits.

First, this uncoupling helps participants focus on their ability to effectively communicate what is on their minds—their desires, feelings, ideas, opinions, notions, requests, and thoughts. Focusing on their communication, rather than focusing on how fluent their talking may be, enables them to speak when they must, need, or want to, without a dependency on fluency being the conduit and/or measure of the effectiveness of their message.

Second, such decoupling not only helps participants shift any and all focus from stuttering/fluency to a focus on changes in communication and supporting variables. Our beliefs and experience indicate that this shift provides an opportunity to strengthen their communicative effectiveness. Such strengthening appears to be—based on the above reported preliminary findings—associated with increased QOL.

### 3.6 QOL and stuttering

As noted above, there is a lack of empirical evidence suggesting that the QOL is reliably improved by treatment that focuses in part, or in whole, on decreasing or modifying stuttered speech (Johnson et al., 2016). Even if such an improvement does occur, there appears to be a relative dearth of published studies on the topic, as well as considerable variability within and across studies. It may be argued, of course, that fluency shaping and/or stuttering modification are not primarily designed to improve the QOL. Rather, the goal(s) of such approaches are designed to correct, cure, or fix the disabling condition of stuttering. As mentioned above, this is a reasonable goal.

However, we would ask what else might such improvements in fluency, modifications of stuttering, and so forth provide the child or adult? Do changes in their fluency/stuttering enhance their communication effectiveness? Do these same individuals who receive FT experience an enhanced QOL? Are these individuals better able to cope with the negative stereotyping often associated with stuttering? Interestingly, it seems reasonable to suggest that the prevailing zeitgeist would answer such questions in the affirmative; however, where are the data to support such admirable outcomes? We would suggest that these questions should probably not be *de facto* answered in the affirmative. Rather, they might better be recognized as open empirical questions that must await answers based on the results of scientific investigation.

## 4 Conclusion

To date, treatments for stuttering have been based on assumptions, beliefs, or notions related to modifying, correcting, curing, or fixing a disabling condition. Notably, in light of the increased understanding within healthcare of the potential negative impacts of ableism, there appears to be a reframing of modification as a positive, healthy strategy that makes communication easier. However, such a treatment approach must give caution to previously documented challenges with regard to transfer and maintenance and implicit messaging. Further, as previously noted, stuttering more easily during daily life imposes a cognitive burden, which limits their ability to focus on communication and, regardless of the intent, suggests that the overt behavior of stuttering needs to, at least, be decreased.

Thus, at present, some researchers and clinicians, the present authors included, have been developing and testing a different, non-ableist approach to the treatment of stuttering. As discussed above, the CT focuses on communication and supporting variables (e.g., education) without targeting the modification of stuttering and/or increasing fluency. This shift in focus requires the adoption of a related but different conceptualization and paradigm for the treatment of stuttering. Perhaps most importantly, this paradigm shift has the potential to impact what is specifically done and/or measured during diagnosis and treatment of stuttering. Clearly, such paradigmatic changes and shifts are seldom easy, quick, or simple.

We recognize the practical complexities of attempting such changes. If and when this approach has been reasonably established as a viable means for treating stuttering in children and adults, its results will need to be compared with treatments that include fluency as a focus, which is the current prevailing practice for stuttering treatment. Such comparisons must first await the reasonably firm establishment of the CT, its assumptions, procedures, results, and tenets.

As of this writing, what seems clear is that comparing, and contrasting two or more therapeutic approaches would and should improve all approaches and offer the public a better service, no matter what service is offered. The clinical studies of CT presented by the authors are preliminary, single-arm clinical efficacy trials. Longitudinal and control-group trials, along with double-arm trials, are warranted but must be conducted taking into account the inherent challenges of examining non-pharmacological treatments using these experimental designs (Hart, 2017). Investigations that contrast the applicability of CT to individuals with co-occurring diagnoses (Blood et al., 2003; Druker et al., 2019; Elsherif et al., 2021; Howell and Davis, 2011; see Briley and Ellis, 2018 and Choo et al., 2020) are underway, as these individuals comprise a notable proportion of our caseload each year (approximately 38% of our 2024 cohort). In fact, the core assumptions of CT permit its expansion to other communication differences, such as autism, cluttering, dyslexia, phonology, etc. Also underway are investigations of the adaptability and efficacy of CT across languages and cultures (e.g., Gruyaert and Lessens, 2023; Węsierska et al., 2024; Choi et al., 2024). As of 2024, CT has been administered in 22 different locations globally [United States (seven locations), the Netherlands (four locations); Ireland (two locations), Israel, Pakistan, Malta, Belgium, Nigeria, Norway, Romania, Uzbekistan, Tanzania] and 11 different languages (English, Dutch, Polish, Maltese, Hebrew, Spanish, Urdu, Romanian, Swahili, Norwegian, and Uzbek). Conducting such comparisons will be neither fast nor easy. However, anything worth achieving is worth working to obtain. As such work is accomplished, the future of stuttering treatment should hold bright promise not only for the public but also for those who provide and study its treatment.

## 5 Call to Action

One final important aspect to consider, whether one implements FT or CT, is the validity of stuttering frequency as a measure of meaningful change. The frequency of stuttering exists on a continuum, varying for each individual daily, monthly, and yearly, and does not accurately capture the impact of the experience. With this one simple yet important adjustment, we can change the conversations about stuttering within the classroom, the clinical environment, the home, and beyond. Teachers can shift their focus to the child’s engagement with them and their peers rather than on how much they are or are not stuttering. Clinicians can strengthen the child’s overall communication skills rather than using the fluency of their speech as an indicator for how effectively they communicate. Caregivers can reinforce their child’s attitude toward communication rather than reminding them to try harder to be more fluent. Public discourse can accurately reflect what stuttering is rather than what it is not, debunking the pervasive misperception that if children are less nervous and more confident, they will speak more fluently.

Thus, with this one simple but important change, we can make significant steps toward ending the stigmatization of stuttering that all too often begins in what should be our safest spaces. Together, with this one change, we can impact countless lives. We ask that all who read this chapter consider supporting us in our Call to Action to no longer use how much a person stutters as an indicator of their eligibility to receive services or their progress or lack thereof, particularly within the school setting, where children and adults are most likely to receive treatment. To learn more about this initiative and join us in our efforts to advocate for this change, stuttering@austin.utexas.edu.

## Author’s Note

The first author is the Founding and Executive Director of the Arthur M. Blank Center for Stuttering Education and Research, a full professor, and a certified speech-language pathologist with more than 30 years of clinical and research experience. The second author stutters openly, is the Associate Director of Grant and Research Development at the Arthur M. Blank Center for Stuttering Education and Research, a parent of someone who stutters openly, and a certified speech-language pathologist with more than 15 years of clinical and research experience. The third author is the Senior Director of Grant and Research Development at the Arthur M. Blank Center for Stuttering Education and Research, a retired speech-language pathologist, and a Professor Emeritus from Vanderbilt University with more than 50 years of clinical and research experience. As noted, the authors have experience developing and/or administering CARE Model treatment with children and adults, which motivates their combined interest in investigating its clinical efficacy.

## Data Availability

The original contributions presented in the study are included in the article/supplementary material, further inquiries can be directed to the corresponding author.

## Footnotes

^1^An RCT is defined as a longitudinal clinical trial wherein participants are randomly assigned to a treatment arm or a second arm (e.g., placebo, control-group, alternative treatment), ideally without knowledge of their assignment (i.e., blinding), and assessed using identical outcome measures [e.g., Food and Drug Administration (FDA), 2019; Capili, 2023; Zabor et al., 2020]. We acknowledge that RCTs of non-pharmacological, experience-based interventions, such as stuttering interventions, are challenging because of difficulties blinding participants and controlling for extra-therapeutic/placebo effects (Hart, 2017; Hart et al., 2008; Whitehead, 2004). Further, treatments that focus on whole-person wellness create an additional challenge in the precise examination of which active ingredients or combinations of ingredients were considered most relevant to each participant.

^2^For the present discussion, communication competence is indexed by the perceptual, Likert-scaled evaluation of the *effectiveness* of communication behaviors within a given situation.

^3^According to the guidelines from the National Institutes of Health [Advisory Committee Subgroup on Individuals with Disabilities of National Institutes of Health, 2022] and the American Psychological Association (American Psychological Association, 2022), an ableist approach to treating someone with a “disability” includes the belief that people with a ‘disability’ are flawed and need to be “fixed.”

^4^This analogy holds more relevance than expected, as there were equally pseudoscientific claims that forced right-handedness in left-handed children was a cure for stuttering (see Kushner, 2012).
